# Statins Improve the Resolution of Established Murine Venous Thrombosis: Reductions in Thrombus Burden and Vein Wall Scarring

**DOI:** 10.1371/journal.pone.0116621

**Published:** 2015-02-13

**Authors:** Chase W. Kessinger, Jin Won Kim, Peter K. Henke, Brian Thompson, Jason R. McCarthy, Tetsuya Hara, Martin Sillesen, Ronan J. P. Margey, Peter Libby, Ralph Weissleder, Charles P. Lin, Farouc A. Jaffer

**Affiliations:** 1 Cardiovascular Research Center, Cardiology Division, Massachusetts General Hospital, Harvard Medical School, Boston, Massachusetts, United States of America; 2 Multimodal Imaging and Theranostic Lab, Cardiovascular Center, Korea University Guro Hospital, Seoul, Republic of Korea; 3 Section of Vascular Surgery, Department of Surgery, University of Michigan, Ann Arbor, Michigan, United States of America; 4 Wellman Center for Photomedicine, Massachusetts General Hospital, Harvard Medical School, Boston, Massachusetts, United States of America; 5 Center for Molecular Imaging Research, Massachusetts General Hospital, Harvard Medical School, Boston, Massachusetts, United States of America; 6 Center for Systems Biology, Massachusetts General Hospital, Boston, Massachusetts, United States of America; 7 Division of Trauma, Emergency Surgery and Surgical Critical Care, Massachusetts General Hospital, Harvard Medical School, Boston, Massachusetts, United States of America; 8 Cardiovascular Division, Department of Medicine, Brigham and Women’s Hospital, Boston, Massachusetts, United States of America; University of Munich, GERMANY

## Abstract

Despite anticoagulation therapy, up to one-half of patients with deep vein thrombosis (DVT) will develop the post-thrombotic syndrome (PTS). Improving the long-term outcome of DVT patients at risk for PTS will therefore require new approaches. Here we investigate the effects of statins—lipid-lowering agents with anti-thrombotic and anti-inflammatory properties—in decreasing thrombus burden and decreasing vein wall injury, mediators of PTS, in established murine stasis and non-stasis chemical-induced venous thrombosis (N = 282 mice). Treatment of mice with daily atorvastatin or rosuvastatin significantly reduced stasis venous thrombus burden by 25% without affecting lipid levels, blood coagulation parameters, or blood cell counts. Statin-driven reductions in VT burden (thrombus mass for stasis thrombi, intravital microscopy thrombus area for non-stasis thrombi) compared similarly to the therapeutic anticoagulant effects of low molecular weight heparin. Blood from statin-treated mice showed significant reductions in platelet aggregation and clot stability. Statins additionally reduced thrombus plasminogen activator inhibitor-1 (PAI-1), tissue factor, neutrophils, myeloperoxidase, neutrophil extracellular traps (NETs), and macrophages, and these effects were most notable in the earlier timepoints after DVT formation. In addition, statins reduced DVT-induced vein wall scarring by 50% durably up to day 21 in stasis VT, as shown by polarized light microscopy of picrosirius red-stained vein wall collagen. The overall results demonstrate that statins improve VT resolution via profibrinolytic, anticoagulant, antiplatelet, and anti-vein wall scarring effects. Statins may therefore offer a new pharmacotherapeutic approach to improve DVT resolution and to reduce the post-thrombotic syndrome, particularly in subjects who are ineligible for anticoagulation therapy.

## Introduction

Due to its sequelae of pulmonary embolism and the post-thrombotic syndrome (PTS), deep vein thrombosis (DVT) causes a substantial burden of cardiovascular morbidity and mortality worldwide, affecting more than 250,000 patients in the United States annually [1]. PTS, a syndrome driven by venous hypertension arising from obstructing thrombi and local vein wall scarring and dysfunction [2–4], occurs more frequently when anticoagulation is subtherapeutic [5,6]. Moreover, up to 50% of DVT patients receiving anticoagulation still develop PTS. Patients with severe PTS can experience debilitating symptoms, such as venous claudication, stasis dermatitis, and skin ulceration, and select cases may even require limb amputation [2,3,7]. Advanced PTS impairs quality of life to the same extent as chronic obstructive pulmonary disease, congestive heart failure, and diabetes [8].

Improving the outcomes of patients with DVT and those at risk for PTS therefore will require new approaches beyond anticoagulation [8]. The primary therapeutic approach to prevent PTS involves strategies to improve DVT resolution or eliminating thrombus burden, particularly for large-vein (e.g., iliofemoral) DVT [9,10]. Given their pleiotropic anti-thrombotic and anti-inflammatory effects beyond their lipid-lowering actions [11,12], 3-hydoxy-3-methyl-glutaryl coenzyme A inhibitors, statins, are an intriguing option to improve DVT resolution and thereby limit PTS. While pretreatment with statins may prevent DVT [13–17], many patients who present with DVT are not taking statins. Furthermore, minimal data exists on whether statins can serve as an effective therapy *after* subjects present with a DVT–a common clinical scenario.

This study evaluated these hypotheses by investigating the *in vivo* time-dependent and dose-dependent effects of daily atorvastatin or rosuvastatin oral therapy initiated either 1 day or 3 days after venous thrombosis (VT) formation, in established, already formed stasis or nonstasis chemical-induced murine VT. We assessed the effects of statins on venous thrombosis burden and vein wall scarring, key drivers of the post-thrombotic syndrome,[2–4] and the fibrinolytic, anticoagulant, antiplatelet, and anti-inflammatory mechanisms of statins involved in VT resolution.

## Methods

### Mouse Cohort

Animal studies were approved and performed in accordance with the Subcommittee on Research Animal Care at Massachusetts General Hospital. Venous thrombosis studies were performed in naïve male 14-week-old C57/BL6 mice weighing 27.3 ± 1.1 grams (N = 282). For all surgical procedures, mice were anesthetized with an intraperitoneal injection of ketamine and xylazine (80/12 mg/kg). Surgical procedures utilized a stereozoom microscope. All mice tolerated the surgical procedures well and were kept warm throughout using a recirculating warm-water blanket. Mice were returned to the animal housing facility once ambulant post-procedure. Mice chow and water were provided *ad libitum*. No qualitative differences in feeding, sleeping, drinking and activity were apparent.

### Statin Therapy

Mice were randomized to treatment groups and started treatment 24 hours after DVT induction and continued until study endpoints (S1 Fig.). All dosages were administrated via oral gavage using a straight 22G feeding needle. The atorvastatin (ATV, MGH Pharmacy) treatment group received a daily dose of 0.38 mg/kg (ATV Low) or 1.14 mg/kg (ATV, based on prior therapeutic dose in mice [18]), dissolved in 0.1 mL of PBS. Rosuvastatin (ROS, MGH Pharmacy) was administered by daily gavage in 0.1mL of PBS at a concentration of 0.28 mg/kg or 0.84 mg/kg (ROS Low and ROS, respectively, at ~25% matched lower doses compared to ATV, due to ROS’ higher potency). The PBS control group received daily oral gavage of 0.1 mL of PBS.

### Assessment of statin and low molecular weight heparin in VT treatment

To compare the magnitude of antithrombotic effect of statins to a conventional anticoagulant, additional mice (N = 32) with stasis IVC VT received subcutaneous low molecular treatment daily (enoxaparin 10 mg/kg SQ) for three days. Control mice received PBS. In addition, evaluation of combination daily statin (atorvastatin 1.14mg/kg or rosuvastatin 0.84 mg/kg) and enoxaparin 10 mg/kg LMWH therapy was examined. To verify the systemic anticoagulant effects of LMWH, factor Xa activity levels were measured in blood samples obtained four hours after the final LMWH injection, and (SensoLyte 520 Factor Xa Assay Kit, AnaSpec Inc.).

### Stasis-induced venous thrombosis

Stasis venous thrombosis was induced following previously published methods [19–22]. Briefly, once anesthetized, male C57/BL6 mice (N = 176) underwent laparotomy, exteriorization of the intestines and exposure of the infrarenal inferior vena cava (IVC). Intestines were kept moist by saline-soaked sterile gauze throughout the procedure. The IVC was then dissected away from the aorta. All visible side branches proximal to the iliac bifurcation were ligated with 7-0 polypropylene sutures. The back branches were left patent. The IVC was then ligated immediately distal to the renal veins with 6-0 polypropylene suture. Exteriorized organs were replaced and the peritoneum was closed using 6-0 polypropylene in a continuous suture, followed by closure of the skin with a 6-0 nylon suture in a simple interrupted pattern. Mice were randomized to various treatment groups 24 hours post-induction of VT.

Mice were sacrificed at day 1, 4, 7, 10, 12 or 21 and the abdominal IVC (infrarenal to the iliac bifurcation) was harvested. The thrombus was carefully removed from the IVC, rinsed in PBS, and weighed. At the time of resection, a white-light micrograph of the thrombus was captured using a Nikon D40 camera (Japan) mounted to the dissection scope. Micrographs were calibrated and used to measure thrombus length. Thrombus mass was defined as thrombus weight divided by thrombus length [22–24]. Venous thrombosis was evident in all mice at day 1 and thereafter.

### Ferric chloride-induced venous thrombosis

Ferric chloride (FeCl_3_)-induced VT was induced in the femoral/saphenous vein [25–27] of male C57/BL6 mice (N = 40; 20 per group). The femoral vein was surgically exposed. A small piece of Whatman #1 filter paper was saturated with 7.5% FeCl_3_ for 1 minute and then applied to the surface of the vein for 3 minutes. After FeCl_3_ treatment, the area was thoroughly rinsed with sterile saline and the incision was closed with a 7-0 nylon suture in a simple interrupted pattern. Venous thrombosis was visible by eye 10 minutes after FeCl_3_ application.

### Confocal intravital molecular-structural imaging of DVT resolution

Statin-induced changes in thrombus burden and thrombus inflammation (macrophages, matrix metalloproteinase (MMP) activity) were measured *in vivo* using molecular-structural intravital microscopy (IVM) of femoral/saphenous VT (N = 24; 12 per group)[27,28]. For IVM molecular imaging of thrombus macrophages and MMP activity at day 4, a macrophage-avid dextranated nanoparticle (CLIO-AF555, 10 mg/kg, Center for Systems Biology Chemistry Core at Massachusetts General Hospital MGH, excitation/emission 555/565nm) and MMP activity sensor (MMP-2,-3,-9, and-13 activatable, MMPSense680, 150 nmol/kg, PerkinElmer, ex/em 680/700nm) dissolved in sterile PBS were injected via tail vein 24 hours beforehand, on day 3. On day 4 fifteen minutes prior to IVM, anesthetized mice were intravenously injected with fluorescent isothiocyanate-conjugated dextran (FITC-dextran, MW: 2,000,000, ex/em 490/520 nm, 0.5 mg in 100 μL PBS, Sigma) to provide angiograms and quantification of venous thrombosis [27].

In addition, serial IVM of thrombus burden was used to quantify statin-driven reductions in thrombus burden dynamically (N = 16 mice; 8 per group). For serial IVM studies at day 2 and day 4 in ferric chloride-induced femoral/saphenous VT, FITC-dextran was injected each time immediately before imaging. Residual FITC-dextran was not present 48 hours after injection, and therefore the thrombus length and volume measures were not confounded by retained FITC-dextran from prior injections delivery [27].

Intravital images were obtained on a custom-built, multiplexed confocal, two-photon microscope system [29]. High-resolution images of the femoral vein were captured at 30 frames/sec utilizing a 30X water-dipping LOMO objective, yielding a field-of-view of 714 μm by 714 μm. Images were generated as the summation of 30 time-averaged frames, and Z-stacks were acquired using a 5 μm step-size up-to depths of 150 μm from the superficial vein surface. Vessels were excited with laser light at wavelengths of 491 nm and 532 nm, generated by a single diode-pumped solid state laser (Dual Calypso, Cobolt AB) and the 635 nm excitation light generated by a diode laser (Radius, Coherent Inc.). FITC-dextran, CLIO-AF555, and MMPSense680 fluorescence were collected through a 509–547 nm bandpass filter, a 573–613 nm bandpass filter, and a 667–722 nm bandpass filter, respectively.

### Polarized microscopy of picrosirius red stained vein wall collagen

Stasis-induced VT sections were stained with picrosirius red (S2365, Poly Scientific) to visualize collagen content in the venous wall in at day 2, 4, 8, 14, or 21 (n = 40; 4 mice per timepoint per group). Polarized micrographs were obtained via an Eclipse 90i microscope (Nikon, Japan) equipped with a color camera and utilizing NIS Elements Software. The vein wall thickness defined by the polarized micrographs were then measured on NIH ImageJ.

### Image analysis

All image analysis utilized ImageJ software (64-bit, 1.47g, NIH, Bethesda, MD,[27]) unless otherwise noted. Briefly, z-stacks covering adjacent healthy vein and the thrombosed area were automatically stitched together in three-dimensions to create one large image stack of the entire thrombosed vein (ImageJ plugin[30]). The target-to-background ratios (TBRs) of CLIO- and MMPSense-enhanced thrombi were calculated from the 40 μm thick mid-lumen volume of z-steps from 20 μm to 60 μm from the surface of the vein. The thrombus TBR for CLIO or MMPSense was defined as: (whole thrombus ROI signal)/(healthy vessel background ROI signal). The background signal was measured on the healthy contralateral veins using a 40 μm thick mid-lumen summation. Whole thrombus ROIs were manually selected from the mid-lumen micrographs on the FITC-dextran angiogram channel. A previous publication reported a high intraclass correlation coefficient for the IVM measurement of thrombus area, and the IVM thrombus length was not statistically different compared to histological-based measurements [27].

### Histology and Immunohistochemistry

Thrombosed veins or resected thrombi were carefully embedded in optimal cutting temperature (OCT) media and flash-frozen using a dry ice/isopentane slurry. 6-μm cryosections were cut for all histological analyses.

FeCl_3_-induced venous thrombosis sections, were stained for the presence of plasminogen activator inhibitor (PAI-1,1:50, sc-8979, Santa Cruz Biotechnology Inc.) and tissue factor (TF, 1:100, ab104513, Abcam). Endogenous peroxidase activity was neutralized with 0.3% hydrogen peroxide treatment in PBS for 30 minutes followed by blocking (3% goat serum, 30 minutes) and primary antibody incubation overnight at 4°C. Immunopositive areas were visualized with Vectastain ABC kit and DAB substrate (Vector Labs). All sections were counterstained with Harris hematoxylin. Sections from 24 animals (12 animals per group) were quantified for each histological analysis. Adjacent sections were stain by hematoxylin-eosin (HE).

### Measurement of total cholesterol, plasma PAI-1, D-dimer, TAT, and fibrinogen levels

At the time of sacrifice, blood was obtained by cardiac puncture with a heparinized syringe, and then centrifuged at 5000 rpm for 5 minutes. Plasma was isolated and stored at −80°C until time of assay. The total cholesterol levels were determined using a commercial colorimetric assay (T-Cho, Wako Chemicals). A PAI-1 ELISA kit (MPAI KT, Molecular Innovations) was utilized to measure plasma levels of total PAI-1. D-dimer (D2D, USCN Life Science Inc.), thrombin-antithrombin (TAT) complexes (IRAPKT175, Innovative Research) and fibrinogen levels (MFBGNKT, Molecular Innovations) were measured using commercially available ELISA kits.

### Immunoblotting

The IVC thrombi were minced, homogenized and extracted with RIPA lysis buffer (Sigma) supplemented with proteinase inhibitors (cOmplete, Roche). Extracted protein concentration was determined by Bradford and after SDS-PAGE and transfer, membrane were probed for the presence of PAI-1 (1:200) and TF (1:1000) using above antibodies. Mac-3 (1:200, sc-19991, Santa Cruz), myeloperoxidase (1:1000, Ab-1, ThermoScientific), Ly6G (1:1000, 551459, BD Pharmigen) and citrullinated histone H3 (CitH3, 1:1000, ab5103, Abcam). Secondary HRP-conjugated goat anti-rabbit (1:1000, sc-2004, Santa Cruz Biotechnology Inc.) and HRP-conjugated goat anti-rat (1:1000, sc-2065, Santa Cruz Biotechnology Inc.) antibodies were utilized where applicable and then detected with ECL substrate (PerkinElmer). The loading control was β-actin (Sigma).

### Measurement of platelet function

In C57/BL6 mice without thrombosis (N = 12 statin, N = 12 control), the effect of 3 days of statin therapy on platelet function was assessed by whole blood impedance aggregometry using the Multiplate platelet function analyzer (Verum Diagnostica Gmbh, [31,32]). Briefly, the Multiplate platelet analyzer is a U.S. Food and Drug Administration (FDA)-approved platelet function analyzer that measures electrical impedance generated by activated platelets attached to two sets of electrodes (for internal control) in a heparinized whole blood sample. The result consists of platelet aggregation curves with arbitrary aggregation units, measured over a 5-minute activation period. Platelet function is quantified as the mean area under the curve (AUC) and expressed in arbitrary units (a.u.).

Whole blood was obtained via cardiac puncture into lithium-heparinized tubes and rested for 30 minutes at room temperature prior to analysis. Platelets were activated with agonists–adenosine diphosphate (ADP, final concentration 6.5 μg/ml), collagen (COL, type 1 Collagen, final concentration 3.2 μg/ml), and arachidonic acid (AA, final concentration 0.5 mM). We also attempted platelet activation with thrombin receptor-activating peptide—6 (TRAP-6, SFLLRN, final concentration 32 μM), but it resulted in little or no activation of murine platelets (data not shown). Consequently, analysis with this agonist was discontinued and results are not reported. All agonists were purchased from the Multiplate manufacturer as part of the clinical standard assay, and all protocols followed manufacturer guidelines.

### Thrombelastography

Thrombelastography (TEG) was performed using the TEG-5000 system (Haemonetics) according to the manufacturer’s instructions. Briefly, citrated whole blood was collected and then rested for 15 minutes prior to assaying. Each sample was activated with kaolin as part of the standard assay. Recorded values were reaction time (r time; time to initial fibrin formation), alpha angle (α-angle; speed of clot build-up indicating interaction between fibrin and activated platelets and thus an indirect marker of platelet function) and maximum clot strength (MA, final product of fibrin and platelet interaction and thus also an indirect marker of clot strength). (N = 3 mice were pooled per group per run). A total of N = 18 C57/BL6 mice without thrombosis (9 atorvastatin and 9 PBS control) were used for 6 separate runs (3 for atorvastatin and 3 for PBS).

### Measurement of bleeding time

The tail vein bleeding time was measured by determining the time required for clotting to occur after a transverse incision was made by an #10 scalpel blade over a lateral vein at a position where the tail was 2.5 mm thick [33]. After the incision, the tail vein was immersed in 37°C PBS in a conical tube. The time from incision to the cessation of bleeding was recorded as the bleeding time.

### Statistical analysis

All results are reported as mean ± SD. Statistical analysis was performed using Prism (ver. 5.0c, GraphPad). Datasets were tested for normality using the Shapiro-Wilk test. If normal, two-group comparisons of parametric data were tested using the two-tailed Student’s t test, while nonparametric data were analyzed with the Mann-Whitney test. Significance between multiple groups was assessed by ANOVA with Dunnett’s test to compare all groups to control. Correlation coefficients were calculated as Pearson correlation coefficients. P values less than 0.05 were considered statistically significant.

## Results

### Statins are effective therapeutic agents for improving the resolution of established venous thrombosis

**Figure 1:**
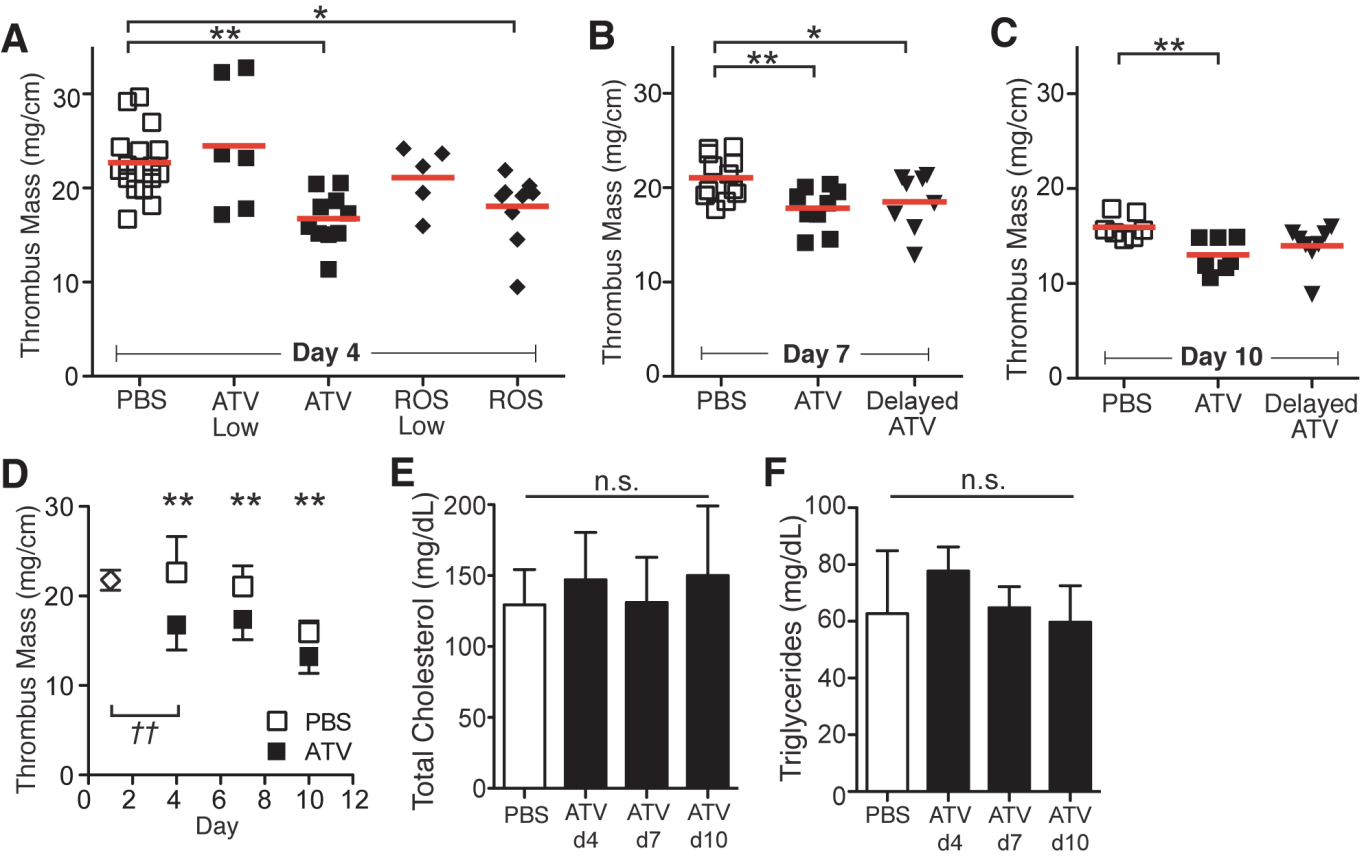
Statin therapy reduces thrombus burden in stasis-induced murine VT. Atorvastatin-treated and rosuvastatin-treated mice animals showed decreased thrombus mass at days 4, 7 and 10 compared to PBS (**A-D**). Atorvastatin therapy started 72 hours (Late ATV) after VT induction showed a similar reduction in thrombus burden at day 7 and day 10 compared to statin therapy started 24 hours after VT induction (upside-down triangle, **B, C**). Analysis of day 1 thrombus mass, before randomization into treatment groups, showed a greater mean thrombus mass compared to day 4 atorvastatin-treated animals (p<0.01) and less than day 4 PBS-treated mice (diamond, **D**). Atorvastatin-treated mice showed similar cholesterol and triglyceride levels compared to PBS-treated mice (**E**, **F**). ATV = atorvastatin; ROS = rosuvastatin; *p<0.05; **p<0.01; *††* p<0.05 between day 4 ATV and day 1 thrombus mass. Mean is marked by red line in scatter plots, and bars represent mean±SD (n = 5–16 animals per group).

**Stasis VT**. At day 0, mice underwent ligation of the IVC to induce stasis VT (Flowchart, S1 Fig.). On day 1, daily statin therapy was initiated on established VT, and its therapeutic effects on thrombus burden were evaluated up to day 10. Mice receiving atorvastatin therapy showed subsequent 26%, 15%, and 18% reductions in mean IVC VT mass over controls at days 4, 7, and 10 after VT induction, respectively (n = 7–16 animals per group, Fig. 1A–1D). Additionally, rosuvastatin treatment showed a significant 25% reduction in day 4 VT mass compared to PBS (Fig. 1A). Low-dose atorvastatin (ATV Low, 0.38 mg/kg) and low-dose rosuvastatin (ROS Low, 0.28 mg/kg) did not significantly reduce VT mass compared to PBS at day 4 (Fig. 1A). Compared to day 1 (diamond, Fig. 1D), atorvastatin-treated mice, but not PBS-treated mice showed a significant reduction in day 4 VT mass. Analyses of the atorvastatin-treated groups showed reduced IVC thrombus burden at day 4, 7 and 10 over control mice, with larger effects present at the earlier time points (Fig. 1D). Delayed initiation of atorvastatin therapy starting at day 3 also reduced VT mass at day 7, but not at day 10 (p = 0.04, day 7 and p = 0.23, day 10 vs. PBS, Fig. 1B and 1C). Total cholesterol and triglyceride levels did not differ significantly across all time points compared to PBS mice (p>0.05, n = 6–8 per group; Fig. 1E and 1F).

To assess the individual and potential additive effects of statin therapy and clinically-employed anticoagulant low molecular weight heparin (LMWH) therapy [34] on established DVT, daily enoxaparin (10 mg/kg) starting at day 1 after DVT formation, without and with daily atorvastatin or rosuvastatin therapy, was studied at day 4 after stasis DVT induction (S2 Fig.). Measurement of IVC thrombus mass revealed similar 26.1%, 20.3% and 20.2% reductions in the atorvastatin, rosuvastatin and LMWH groups, respectively (p<0.001 ATV, p = 0.004 ROS, and p = 0.003 LMWH, vs. PBS, n = 8–16 per group). The combination of LMWH with either atorvastatin or rosuvastatin also significantly reduced IVC thrombus burden (p<0.05, ATV+LMWH or ROS+LMWH vs. PBS, n = 7–16 per group), but to a similar extent as LMWH alone or atorvastatin alone (p>0.05; S2 Fig.). Increasing the LMWH dose (3x = 30 mg/kg) or increasing the atorvastatin dose (5x = 5.7 mg/kg) did not reduce thrombus mass further at day 4 (n = 6 per group, p>0.05, data not shown). LMWH and ATV+LMWH therapy significantly decreased factor Xa (FXa) activity by 55.5±5.8% and 33.7±9.4%, respectively (p<0.05, S2 Fig.B, n = 8 animals per group). There was no significant difference in FXa activity levels between statin only- and PBS-treated animals (p>0.05, S2 Fig.B).

**Figure 2:**
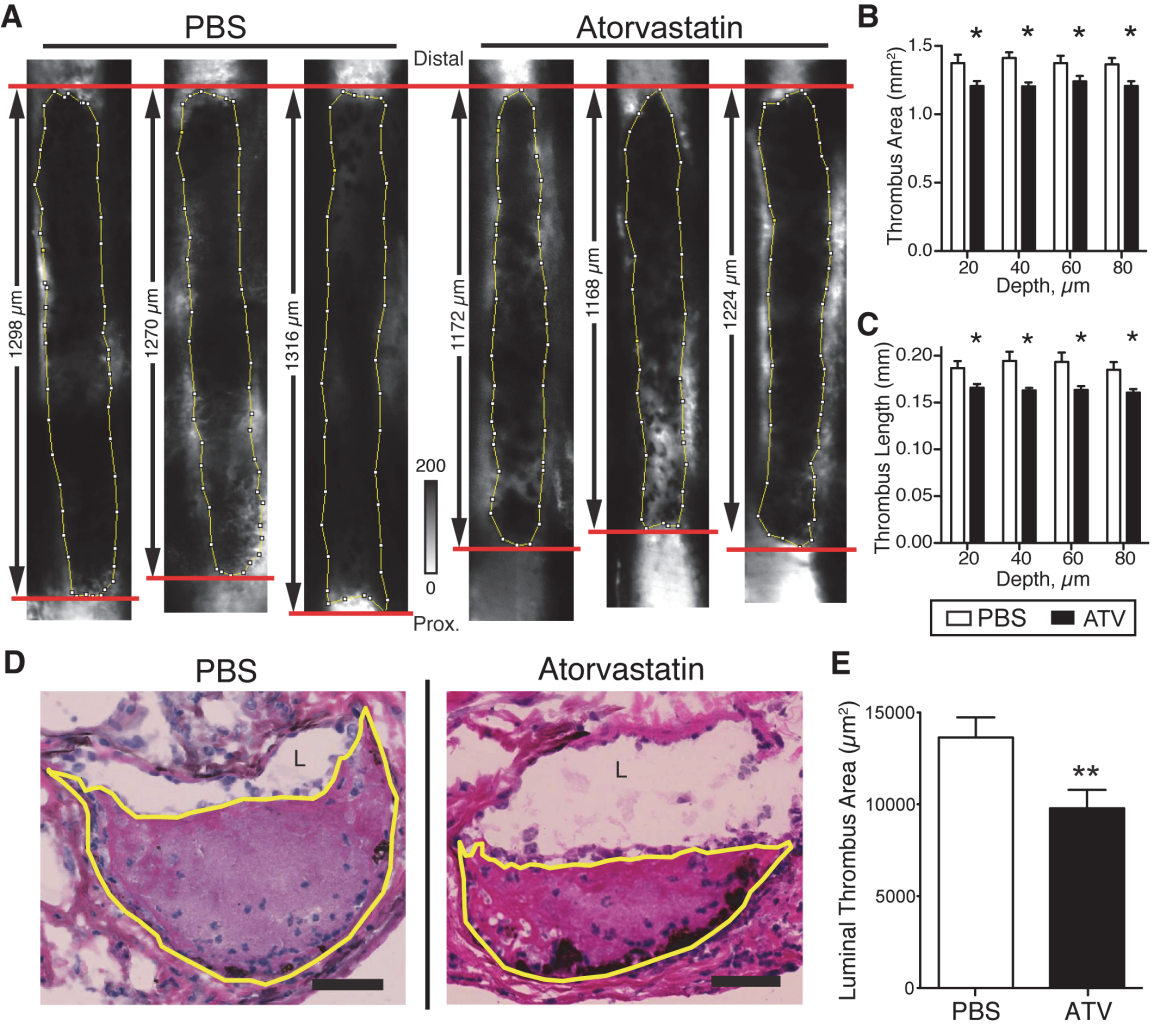
Statins reduce thrombus burden in non-stasis murine DVT. Thrombi were visualized on IVM via a FITC-dextran angiogram as hypointense areas in the vein (**A**). Thrombus area and length measurements at 20, 40, 60, and 80 μm below the superficial wall-lumen interface showed a decrease in thrombus burden in atorvastatin-treated animals vs. PBS (**B, C**). Representative H&E axial sections showed a decrease in luminal thrombosis area in atorvastatin-treated animals compared to PBS (**D, E**). Thrombus outlined in yellow (**D**). ATV–atorvastatin. *p<0.05; ** p<0.01. Bars represent mean±SD of n = 12 animals per group. Scale bars, 25 μm.

**Non-stasis, ferric chloride-induced VT**. Further experiments evaluated the effects of atorvastatin on resolution of DVT induced by topical perivenous application of ferric chloride [25–27]. *In vivo* structural-molecular confocal fluorescence intravital microscopy (IVM) comprehensively assessed thrombus burden, inflammation, architecture and resolution in mouse femoral DVT [27]. Confocal IVM micrographs of FITC-dextran showed thrombi as hypointense regions within the vein lumen. IVM depicted thrombus morphological features, such as area and length, at defined depths below the vein wall-lumen interface (Fig. 2A). Quantitative IVM analyses demonstrated that statin therapy reduced femoral DVT area and length at day 4 by 7–16% (12.5±3.1%, n = 12 animals per group; Fig. 2B and C). Analysis of axial histological sections of the thrombosed femoral vein further assessed thrombus burden (Fig. 2D). Statin treatment resulted in a mean 28.3% reduction in luminal thrombus burden compared to PBS-treated group (0.98±0.41 × 10^4^ μm^2^ thrombus area, vs. PBS group, 1.36±0.46 × 10^4^ μm^2^, p = 0.01, Fig. 2E). Compared to histological assessment, confocal IVM underestimated statin-mediated reductions in thrombus burden in murine femoral vessels, due to a light attenuation-based limitation to image the thrombus at depths below 150 μm. Accordingly, all IVM quantitative analyses used a mid-luminal thrombus volume from 20 μm to 60 μm below the superficial vein wall–lumen interface was utilized, as previously described.[27]

**Figure 3:**
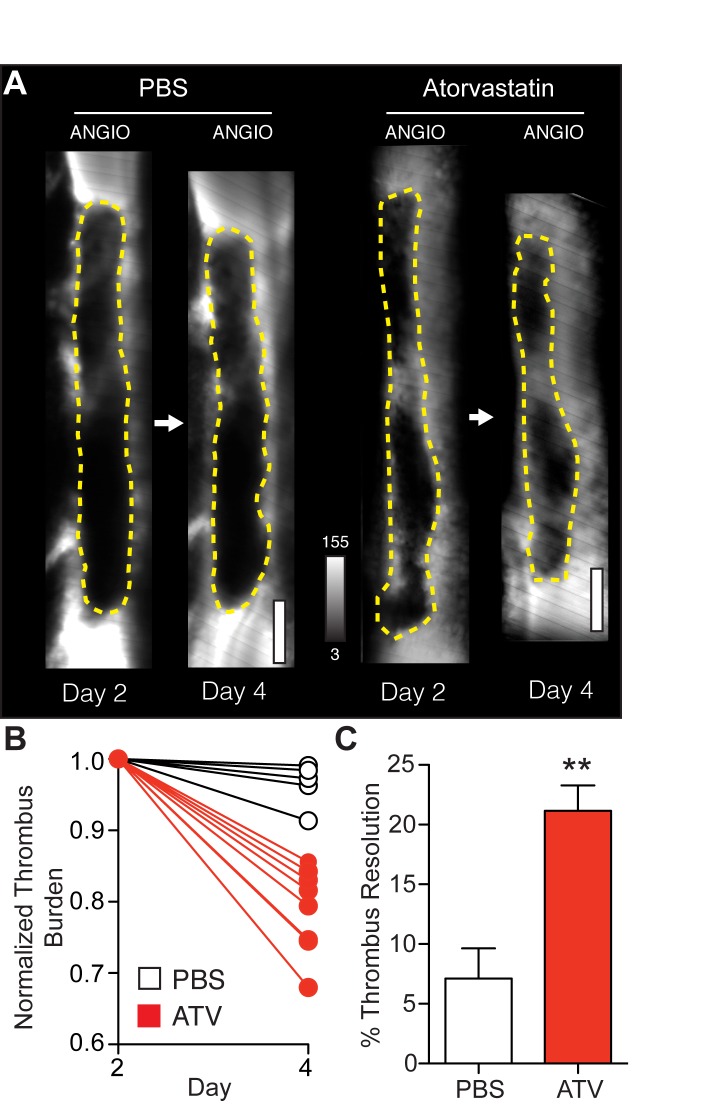
Early venous thrombus resolution is accelerated in atorvastatin-treated animals. Representative serial IVM angiograms at days 2 and 4 post-DVT induction are shown in PBS- and atorvastatin-treated animals with non-stasis VT (**A**). Atorvastatin-treated animals showed greater thrombus resolution at day 4 (**B**, **C**). ATV–atorvastatin. **p<0.01. Bars represent mean±SD of 5–6 animals per group. Scale bars, 200 μm.

To quantify the effects of statin therapy on the rate of thrombus resolution from day 2 to day 4 post-induction of DVT, serial IVM of non-stasis induced VT was performed in another cohort of animals (N = 16 mice, Fig. 3). FITC-dextran angiograms were collected from the same mouse at days 2 and 4. Atorvastatin-treated animals showed a 66.3% increase in thrombus resolution (change in thrombus area from day 2 to day 4) compared to PBS-treated animals (Fig. 3B and 3C, p<0.05 vs. PBS).

### Statins induce pro-fibrinolytic, anti-coagulant, and anti-platelet effects in established murine venous thrombosis

**Figure 4:**
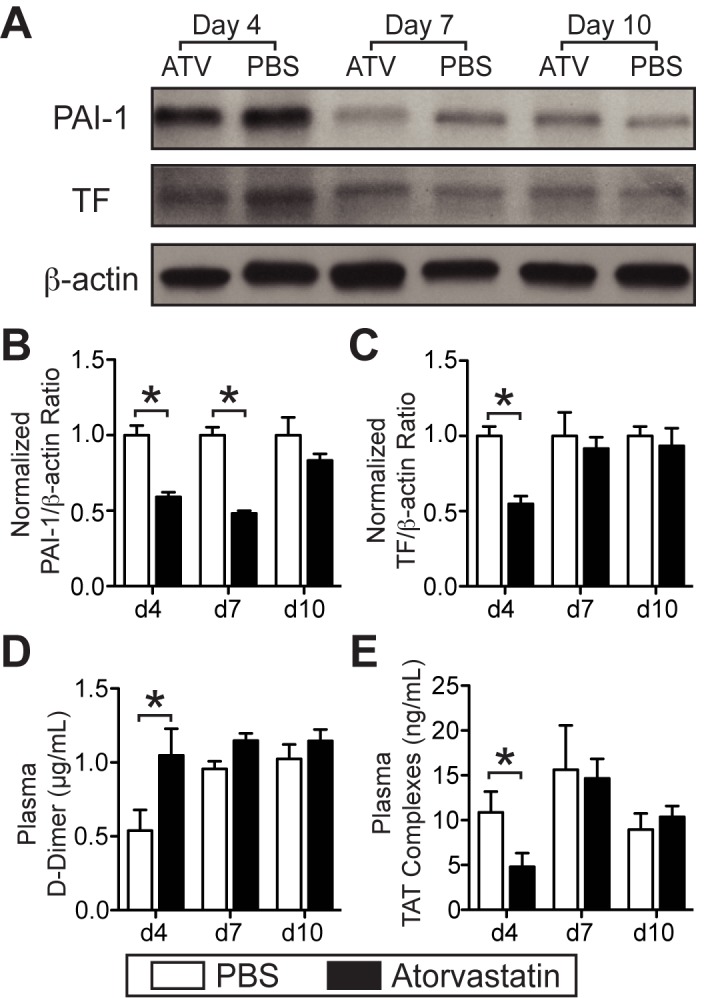
Atorvastatin enhances fibrinolysis and suppresses coagulation in murine VT at early timepoints. Representative immunoblot of PAI-1, TF and loading control β-actin from day 4, 7, and 10 stasis-induced VT is shown (**A**). Immunoblot analysis shows a reduction in the expression of PAI-1 at days 4 and 7 in atorvastatin-treated mice compared to PBS-treated mice (**A**, **B**). Immunoblot analysis also shows a reduction in the day 4 expression of thrombus TF (**A, C**). At day 4, plasma D-dimer levels were higher (**D**), and levels of plasma TAT complexes were decreased in atorvastatin-treated vs. PBS-treated mice (**E**). ATV–atorvastatin; PAI-1–plasminogen activator inhibitor-1; TF–tissue factor. *p<0.05. Bars represent mean±SD. Immunoblots, n = 5–8 per group.

**Pro-fibrinolytic effects**. To explore mechanisms underlying the observed reductions in thrombus burden in statin-treated groups, we investigated multiple putative anti-thrombotic effects of statins. As endogenous fibrinolysis contributes critically to venous thrombus resolution [35], we measured plasma levels of D-dimer, a fibrin degradation product that serves as a systemic marker of fibrinolysis. We also measured the expression of thrombus PAI-1, in statin-treated and PBS-treated groups across days 4, 7 and 10 stasis-induced VT. Atorvastatin decreased thrombus PAI-1 expression in the statin group at days 4 and 7 compared to PBS (p = 0.03 vs. PBS; Fig. 4A and 4B), indicating a profibrinolytic effect of statin therapy. Expression levels of thrombus PAI-1 in statin and PBS groups were similar by day 10. Thrombus PAI-1 expression was also decreased in non-stasis induced VT in atorvastatin-treated mice (14.8±14.0% vs. PBS 30.7±16.0%, p = 0.02; S3 Fig.). Plasma PAI-1 levels trended lower in the statin group at day 4 (ATV, 11.7±5.2 ng/mL; PBS, 14.4±4.5 ng/mL; p = 0.295). Plasma levels of D-dimer were doubled at day 4 in stasis VT animals treated with atorvastatin (Fig. 4D, p<0.05 vs. PBS), consistent with an early profibrinolytic effect of statins. At days 7 and 10, no significant differences in D-dimer levels were found between statin- and PBS-treated animals (Fig. 4D, p>0.05).

**Anticoagulant effects**. To examine the effects of statins on anticoagulant activity in established VT, we analyzed the expression of thrombus TF, the primary initiator of the extrinsic coagulation pathway, as well as the levels of plasma TAT complexes, a marker of thrombin generation. Atorvastatin therapy decreased TF expression in day 4 IVC thrombi (p = 0.03 vs. PBS, Fig. 4A and 4C). Thrombus TF expression was also decreased in non-stasis induced VT in atorvastatin-treated mice (TF-positive area 10.1±4.7% vs. PBS 31.0±16.9%, p = 0.01; S3 Fig.). Plasma TAT levels were also reduced in day 4 statin-treated animals, indicating reduced systemic thrombin generation (Fig. 4E). Plasma TAT levels at days 7 and 10 were not significantly different. Prothrombin time (PT) and partial thromboplastin times (PTT) measured in day 4 animals without VT were similar in statin and PBS groups (PT: 10.5±0.8 seconds ATV vs. 9.90±0.3 seconds PBS, p = 0.4906; PTT: 62.0±8.2 seconds ATV vs. 68.3±4.2 seconds PBS, p = 0.4960).

**Antiplatelet effects**. Recent studies demonstrate a pivotal role of platelets in the genesis and propagation of DVT [36,37], and in the prevention of recurrent clinical DVT [38]. To determine if antiplatelet effects of statins pertain to murine VT resolution, platelet aggregation was measured in whole blood samples using impedance aggregometry (3 mice pooled for each of n = 5 experiments per group). Whole blood from C57/BL6 treated with atorvastatin for 3 days showed significantly reduced ADP-induced platelet aggregation (p = 0.03, Table 1 and S4 Fig.) and AA-induced platelet aggregation (p = 0.01). A nonsignificant reduction in collagen-induced platelet aggregation was observed (p>0.05). Statins did not significantly change platelet count, or plasma calcium levels (all p>0.05). Atorvastatin-treated and PBS-treated groups had similar monocyte (ATV 0.23±0.07 vs. PBS 0.30±0.10 10^3^ cells/μL; p>0.05;), polymorphonuclear neutrophil (ATV 0.67±0.13 vs. PBS 0.83±0.09 10^3^ cells/μL, p>0.05;), and total leukocyte (ATV 3.0±0.70 vs. PBS 2.8±0.66 10^3^ cells/μL, p>0.05;) counts in peripheral blood.

**Table 1:** Differences in platelet aggregation using whole blood impedance aggregometry in experimental groups.

Aggregation A.U.C. (a.u.)	PBS	Atorvastatin	p-value
ADP (6.5 μM)	465.5±145.6	230.1±119.3	0.03
AA (0.5 mM)	576.1±68.7	389.4±79.9	0.01
Collagen (3.4 μg/mL)	586.5±47.6	539.7±54.2	0.26
Platelet count (10^3^/μL)	425.2±87.5	416.6±96.4	0.94
Plasma Ca^++^ (mg/dL)	9.4±0.1	9.6±0.7	0.66

ADP = adenosine diphosphate; AA = arachidonic acid; A.U.C = area under the curve; a.u. = arbitrary units

To assess the integrative effects of atorvastatin therapy on clot formation and integrity, TEG measurements of kaolin-activated whole blood were analyzed (n = 18, 9 per group). TEG measures the viscoelastic properties of clotting of whole blood and reflects platelet, coagulant, and fibrinolytic activities [39], and has been used to understand venous thrombogenesis in patients [40,41]. Common TEG protocols mimic slower venous flow rates, and serve to assess the coagulation state prevailing in clinical and experimental VT. The TEG parameters of reaction (R) time (time until first sign of clotting), clot formation (K) time, clotting rate or rate of fibrin crosslinking (α-angle), and strength of clot (MA) were recorded. Whole blood of atorvastatin-treated mice demonstrated significantly longer R and K times, and lower α-angle and MA values, indicating weaker clotting activity (Table 2). Atorvastatin-treated mice exhibited a small 7.5% increase in fibrinogen plasma concentrations (Table 2, p = 0.01 vs. PBS). They also exhibited longer tail-vein bleeding times, indicating reduced blood clotting (49.8±2.3 seconds vs. PBS 44.0±2.6 seconds, p<0.05, n = 8 animals per group).

**Table 2:** TEG parameters of pooled whole blood from experimental groups.

TEG Parameter	PBS	Atorvastatin	p-value
R (seconds)	4.0±0.8	10.7±2.8	0.02
K (seconds)	1.0±0.3	3.5±1.4	0.04
α-angle (degrees)	75.7±3.1	50.3±11.8	0.02
Maximum amplitude (mm)	72.9±1.4	65.6±3.8	0.04
Fibrinogen (mg/mL)	1.46±0.1	1.58±0.1	0.01

**Figure 5:**
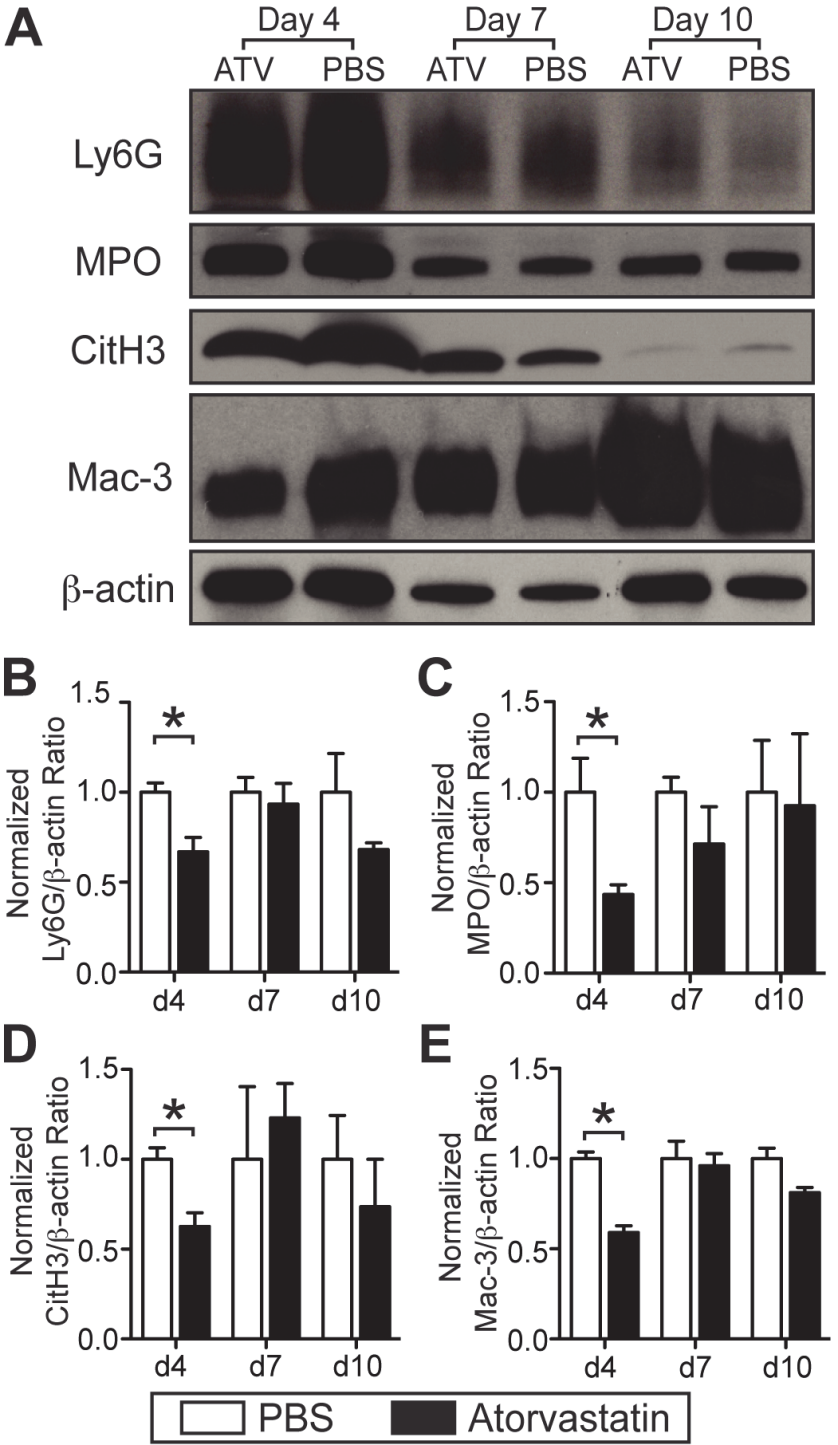
Atorvastatin reduces thrombus inflammation in murine VT at early timepoints. Representative immunoblot of neutrophil markers Ly6G and MPO, NET marker CitH3 and macrophage marker Mac-3 from days 4, 7, and 10 stasis-induced VT is shown (**A**). Immunoblot analysis of stasis-induced VT shows a reduction in all inflammation markers at day 4 in atorvastatin-treated animals. No significant difference in days 7 and 10 were found between statin and PBS groups. Mac-3 expression increased from day 4 to 10, while the other markers decreased (**A**). *p<0.05. Bars represent mean±SD. Immunoblots, n = 5–8 per group.

**Anti-inflammatory effects**. The inflammatory response modulates DVT resolution.[7,27,42] As statins exert multiple anti-inflammatory actions,[11] we further examined the effects of statins on levels of thrombus macrophages, neutrophils, and markers of neutrophil extracellular traps (NETs), such as citrullinated histone H3.[43] Immunoblot analyses of extracted thrombus protein was use to compare atorvastatin- and PBS-treated animals at 4, 7, and 10 days post-stasis induced VT. Immunoblot analysis of macrophages[44] in stasis-induced IVC thrombi demonstrated a 41.1% reduction in thrombus Mac-3 levels in atorvastatin-treated animals at day 4 (p = 0.028, Fig. 5A and 5B), but non-significantly differences at days 7 and 10 compared to PBS (p>0.05). Similar day 4 reductions were also seen in neutrophil levels using myeloperoxidase (MPO) and Ly-6G markers (p<0.05; Fig. 5A, 5C and 5D). Citrullinated histone H3 (CitH3), a marker of NETs, expression levels were also reduced in atorvastatin-treated animals at day 4 (p<0.05; Fig. 5A and 5E). Both neutrophil and CitH3 markers exhibited similar protein levels in days 7 and 10 in statin- and PBS-treated mice (p>0.05; Fig. 5).

Reduction in thrombus inflammation by statins was also assessed *in vivo* in non-stasis VT using intravital microscopy (IVM)-based molecular imaging of FeCl_3_-induced femoral VT (n = 12 per group). Macrophage content and MMP activity, depicted by molecular imaging reporters CLIO-AF555 and activatable MMPSense680 respectively,[27,44] localized within and along the edges of the thrombus (S5 Fig. A,C). CLIO-AF555 and MMPSense680 produced negligible signal in contralateral sham-injury areas, which served as the background area for calculation of the target-to-background ratios (TBRs). Macrophage and MMPs TBRs, calculated from an averaged mid-luminal thrombosis volume, were significantly lower in atorvastatin-treated animals compared to PBS-treated animals at day 4 (macrophage (MAC) TBR 1.74±0.53 vs. control 3.01±0.99, p = 0.0001; and MMP activity TBR 1.30±0.31 vs. control 1.75±0.54, p = 0.01, respectively; S5 Fig. B,D). Whole-thrombus macrophage content and MMP activity correlated strongly in both atorvastatin- and PBS-treated animals, with statin-treated animals exhibiting lower inflammation measures (r = 0.70, p = 0.0002; S5 Fig. E).

*Ex vivo* fluorescence microscopy of axial sections of the femoral vein further evaluated the anti-inflammatory effects of statin treatment on murine VT. Statin treatment decreased thrombus macrophage accumulation and MMP activity in day 4 nonstasis VT of atorvastatin-treated animals (CLIO-AF555/MAC percent positive area, 22.6±15.0%, atorvastatin, vs. PBS 47.4±16.2%, p<0.0001; S6 Fig. A,B). MMP activity analyses yielded similar findings (MMPSense680 percent positive area, 15.2±6.2%, atorvastatin, vs. control 27.9±10.6%, p = 0.002; S6 Fig. C,D).

### Statin therapy reduces vein wall scarring in stasis VT

**Figure 6:**
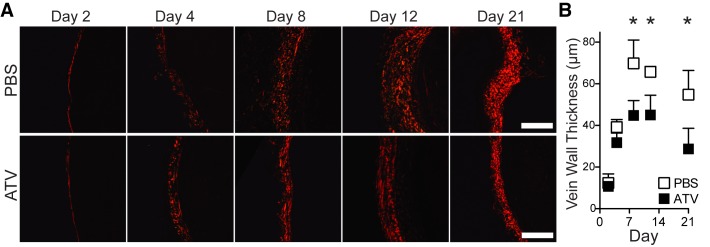
Atorvastatin reduces venous wall injury in stasis-induced murine IVC VT. Representative polarized micrographs of Picrosirius red-stained sections from days 2, 4, 8, 12, or 21 VT show vein wall collagen (red fibers) from atorvastatin- and PBS-treated animals. IVC vein wall collagen thickness was significantly reduced in atorvastatin-treated animals at days 8, 12 and 21. Error bars represent SD. *p<0.05.

Vein wall scarring during DVT formation and resolution may cause valvular incompetence, venous reflux, and venous hypertension, and thereby promote the development of PTS [2–4]. Strategies that reduce DVT-induced vein wall scarring could therefore reduce the incidence of severity of PTS. To investigate the effects of atorvastatin on vein wall fibrosis and injury, IVC sections were analyzed for collagen and vein wall thickness, a surrogate for vein wall scarring. Sections were assessed using picrosirius red stain. Polarized micrographs of VT sections stained by picrosirius red showed collagen vein wall thickness in atorvastatin-treated animals was similar to PBS at days 2 and 4 (p>0.05; Fig. 6), but by days 8, 12 and 21, atorvastatin treatment durably and significantly reduced vein wall scarring compared to PBS-treated animals (43.9%, 39.0% and 47.6% reductions at day 8, 12, and 21 respectively vs. PBS, p<0.0001; Fig. 6).

## Discussion

This murine study found that statin treatment reduced thrombus burden and vein wall injury in established VT, without requiring statin pretreatment prior to VT formation. Statins inhibited several hemostatic functions, promoted a pro-fibrinolytic and anti-thrombogenic state, and decreased VT-driven vein wall scarring. The overall findings show that atorvastatin or rosuvastatin, initiated 1–3 days *after* VT formation, improves VT resolution in both stasis and non-stasis murine VT, and thus may offer a translatable approach to reduce the incidence and severity of the DVT-driven post-thrombotic syndrome.

Statin therapy reduced thrombus burden and accelerated clot resolution, with greater effects in relatively younger VT (Figs. 1,2 and 3). Mechanistically, statins induced several favorable anti-thrombotic effects that pertain to improvement in VT resolution observed in this study. Statin therapy reduced thrombus levels of the fibrinolytic inhibitor PAI-1 and thrombus levels of the coagulation cascade initiator TF, consistent with and extending *in vitro* studies [12,45]. Separately, decreases in either PAI-1 or TF can promote net fibrinolysis of both venous and arterial thrombi [46]. Mechanisms of statin-induced TF inhibition involve the prevention of isoprenoid intermediate synthesis and the inhibition of geranylgeranylation of components of the Rho/Rho kinase pathway *in vitro* [47,48]. The reduced Rho family activation lowers the expression of NF-κB, a key transcription factor that positively regulates TF in VT. In addition, statins promote the expression of Kruppel-like factor 2 (KLF2), a positive regulator of eNOS and thrombomodulin and an inhibitor of TF and PAI-1 [49,50]. Recent experimental observations show that myeloid TF actively orchestrates the formation of VT, with statins inhibiting TF-dependent activation of coagulation in a hypercholesterolemic milieu [37,51]. Therefore, reductions in thrombus PAI-1 and TF expression may be operative in the salutary VT benefits conferred by statins.

Platelet activity and recruitment also support DVT formation and are important in venous thrombogenesis and thrombus extension [36,37]. Clinical and experimental studies demonstrate that statins limit cyclooxygenase-1 (COX-1) activation and augment nitric oxide synthase (NOS), serving to reduce platelet activation and aggregation [52]. Atorvastatin can inhibit platelet α_IIb_β_3_ activation and P-selectin expression in mice even in the absence of endothelial NOS [53]. These studies support our findings that statins reduce ADP-driven and AA-driven platelet aggregation in whole blood of atorvastatin-treated mice. TEG studies demonstrated decreased clot formation and clot stability in the statin group, consistent with statin inhibition of coagulation and platelet activation.

This study also investigated effects of statin therapy on the local inflammatory response in established DVT. At early VT timepoints such as day 4, statin therapy reduced VT macrophage, neutrophil and NET levels compared to controls. Molecular imaging of VT inflammation in nonstasis VT further revealed that statin therapy reduced *in vivo* thrombus macrophage and MMP activity at day 4. Although statin therapy reduced thrombus inflammation, which might impair thrombus resolution [20–22,54,55], we found reduced thrombus burden and improved thrombus resolution in murine VT (Figs. 1,2, and 3), demonstrating that the antithrombotic effects of statins predominate over its anti-inflammatory effects.

Vein wall scarring was durably reduced to day 21 in DVT mice that received statin therapy. Vein wall injury due to VT-induced inflammation and prolonged stasis results in stiffened veins and dysfunctional valves, which promote venous hypertension and PTS [2–4]. While beyond the scope of this study to identify specific mechanisms underlying statin-mediated reductions in vein wall injury, reductions in inflammatory neutrophil and monocyte/macrophage influx and MMP activity may reduce the production of profibrotic factors, serine elastases, MMPs and cathepsins, that contribute to vascular remodeling and the generation of collagen [20–56]. Overall the results demonstrate that statins reduce experimental vein wall injury while also lessening thrombus burden.

This study provides the novel evidence that statins can effectively improve the resolution of established VT, without requiring statin pretreatment prior to VT formation. While pretreatment studies of statins may *prevent* the formation of VT in humans [13–15] and mice [16,17], pretreatment studies do not apply to subjects that present with established DVT, as most such patients are statin-naïve [57]. As routine oral anticoagulation (e.g. warfarin) does not eliminate the risk of PTS [5,6], the results herein, coupled with the excellent safety profile of statins, offers impetus to investigate adjunctive statin therapy in anticoagulated subjects with DVT.

While the focus of this study was to assess the independent effects of statins on established VT, we also compared the effects of statin and anticoagulant therapy alone, and in combination. Statins reduced VT burden to a similar extent as anticoagulation with enoxaparin; the effects of statins, unlike enoxaparin, were not associated with reductions in FXa levels. Mice commonly require supratherapeutic human doses of enoxaparin to achieve anticoagulation (for example 10 mg/kg/day in this study compared to 2 mg/kg/day in clinical subjects [58–60]). Furthermore, most studies have required pre-treatment or peri-DVT initiation of treatment to demonstrate a therapeutic anticoagulant effect. We did not observe an additive benefit of combining both therapies in established murine stasis DVT, possibly related to the relative resistance of murine DVT to enoxaparin, and/or to heightened sensitivity of murine DVT to statins, and/or possible fundamental limits to thrombus reductions that are achievable in the murine stasis IVC model [61].

Nonetheless, statin therapy may be a clinically viable strategy to improve DVT resolution in patients, especially those who are ineligible for anticoagulation–such as those with ongoing bleeding or post-operative from high-risk surgery, or those with prohibitively high hemorrhage risk if anticoagulated. Patients unable to be anticoagulated are at particularly high risk for developing PTS [5,6]. While tens of thousands of DVT patients with contraindications to anticoagulation each year may receive IVC filters to prevent thromboembolism [62], these patients do not routinely receive thrombus-directed therapies to improve DVT resolution and limit PTS. The anti-thrombotic actions of statins observed here could ultimately prove useful in improving DVT resolution and reducing PTS symptoms and incidence in those unable to be anticoagulated.

This study has limitations. Direct effects of statins on PTS could not be tested, as murine models of PTS do not currently exist. As the extent of thrombus burden determines the degree of venous obstruction and vein wall injury, key pathophysiological drivers of PTS [3,4], statins could reduce the severity and incidence of PTS, both through short-term reductions in thrombus burden as well as through durable reductions in vein wall scarring. This investigation did not test the efficacy of initiating statin therapy beyond day 3 after VT induction, but as many patients could present within 3 days of DVT symptoms, this treatment window had clinical relevance. Other statins may improve the resolution of established VT; atorvastatin and rosuvastatin were chosen for this study given their high potency and established effectiveness in other clinical conditions.

In conclusion, statin therapy improves VT resolution of established already formed VT, in concert with profibrinolytic, anticoagulant, antiplatelet, anti-inflammatory, and anti-vein wall scarring effects. These results support statins as a clinically testable therapy to improve the resolution of DVT to potentially mitigate or prevent PTS, particularly in subjects unable to receive anticoagulation.

## Supporting Information

S1 Fig Scheme of experimental groups utilized for in vivo studies in IVC stasis-induced VT (upper panel) and intravital morphological analysis of femoral ferric chloride/nonstasis-induced VT (lower panel). For control groups, statin therapy was replaced by PBS administration. ATV = atorvastatin, LMWH = low molecular weight heparin, ROS = rosuvastatin.
(TIF)

S2 Fig Combination statin and LMWH therapies show significant reduction in thrombus burden in stasis-incuded VT compared to PBS-treated animals. Low molecular weight heparin (LMWH) only, and combination atorvastatin+LMWH (ATV+ROS) and rosuvastatin+LMWH (ROS+LMWH) have similar thrombus masses at day 4 (**A**).PBS, ATV and ROS only groups are included from Fig. 1A for ease of comparison to LMWH groups. LMWH- and ATV+LMWH-treatment showed reduced Factor Xa activity compared to PBS (**B**). Atorvastatin alone nonsignificantly reduced Factor Xa activity (**B**). *p<0.05.
(TIF)

S3 Fig Atorvastatin decreases PAI-1 and TF in ferric chloride non-stasis induced VT. Immunohistochemical analysis of thrombosis sections shows a decrease in thrombus PAI-1 (**A**, **B**) and TF (**C**, **D**) in atorvastatin-treated mice compared to PBS-treated mice at day 4. Yellow dashed box marks the zoomed inset area. ATV = atorvastatin. *p<0.05; ** p<0.01. Bars represent mean±SD of n = 12 mice per group. Scale bars, 25 μm.
(TIF)

S4 Fig Platelet function assessed by whole blood impedance aggregometry, 3 days after atorvastatin or PBS treatment. Atorvastatin (red line) decreased platelet aggregation compared to PBS-treated mice (black line). Adenosine diphosphate (ADP; **A**), arachidonic acid (AA; **B**) and collagen (**C**) were used to stimulate activation of platelets in pooled whole blood samples. Thick solid line and thin lines depict the mean and its SD, respectively.
(TIF)

S5 Fig Atorvastatin reduces thrombus inflammation in non-stasis murine DVT. Macrophages (**A**) and MMP activity (**C**) were visualized in DVT 24 hours after intravenous injection of CLIO-AF555 (green) and MMPSense680 (red), respectively (**A**, **C**). Thrombus macrophage content (**B**) and MMP activity (**D**) were reduced in atorvastatin-treated animals compared to PBS. In vivo macrophage content and MMP activity correlated well in both statin-and PBS-treated animals (**E**). MAC—macrophage; MMP—matrix metalloproteinase, TBR, target-to-background ratio. *p<0.05; **p<0.01. Mean is marked by red line in scatter plots of 12 animals per group. Scale bars, 200 μm.
(TIF)

S6 Fig Representative fluorescence micrographs of axial sections from thrombosed veins of atorvastatin- and PBS-treated animals in day 4 non-stasis VT. FM shows reduced macrophage content (**A**, **B**; green) and MMP activity signals (**C**, **D**; red) in statin-treated compared to PBS-treated animals. Bars represent mean±SD of 12 animals per group, average of three section per animal. Scale bar, 25 μm.
(TIF)
