# Supplementary figures and images for: Statins Improve the Resolution of Established Murine Venous Thrombosis: Reductions in Thrombus Burden and Vein Wall Scarring
Source: PLoS One. 2015 Feb 13;10(2):e0116621. doi: 10.1371/journal.pone.0116621 (PMC4334538; doi:10.1371/journal.pone.0116621)

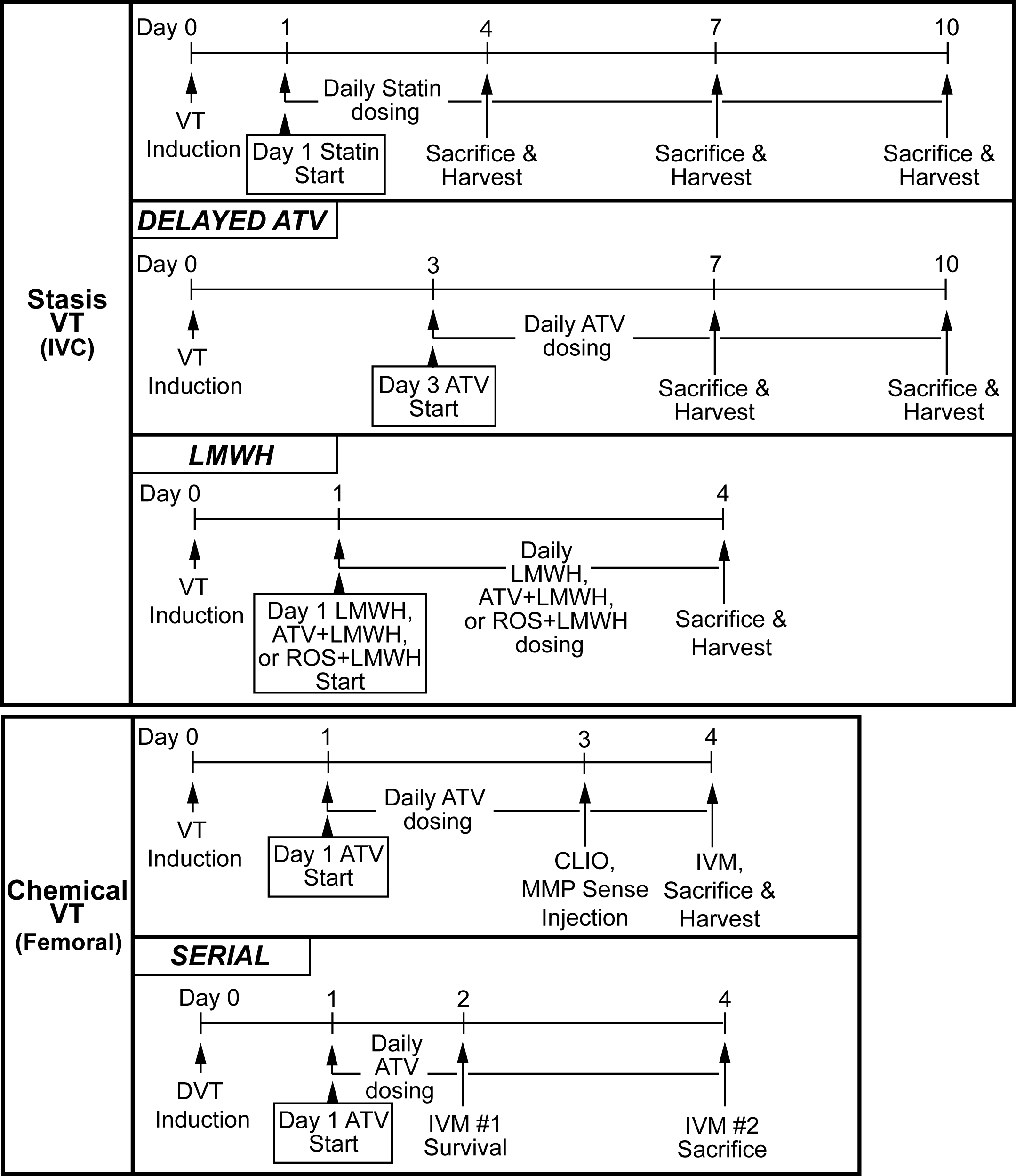

Supplement: S1 Fig — For control groups, statin therapy was replaced by PBS administration. ATV = atorvastatin, LMWH = low molecular weight heparin, ROS = rosuvastatin. (TIF) [file pone.0116621.s001.tif]

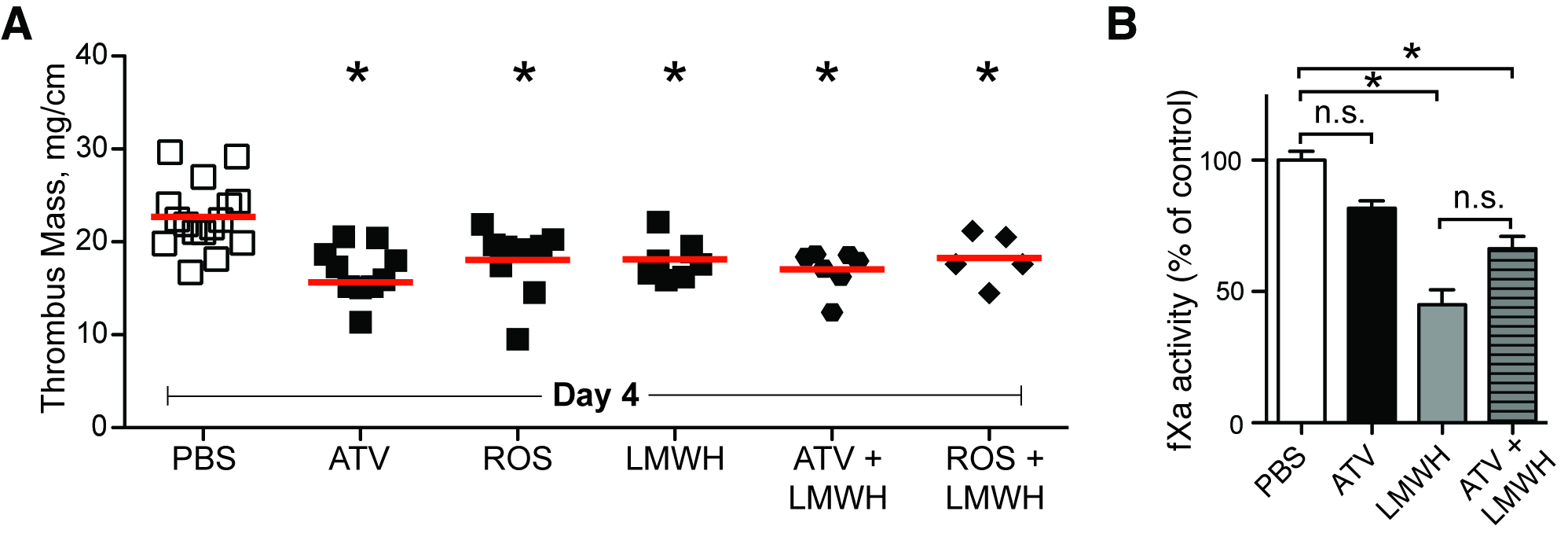

Supplement: S2 Fig — Low molecular weight heparin (LMWH) only, and combination atorvastatin+LMWH (ATV+ROS) and rosuvastatin+LMWH (ROS+LMWH) have similar thrombus masses at day 4 (A).PBS, ATV and ROS only groups are included from Fig. 1A for ease of comparison to LMWH groups. LMWH- and ATV+LMWH-treatment showed reduced Factor Xa activity compared to PBS (B). Atorvastatin alone nonsignificantly reduced Factor Xa activity (B). *p<0.05. (TIF) [file pone.0116621.s002.tif]

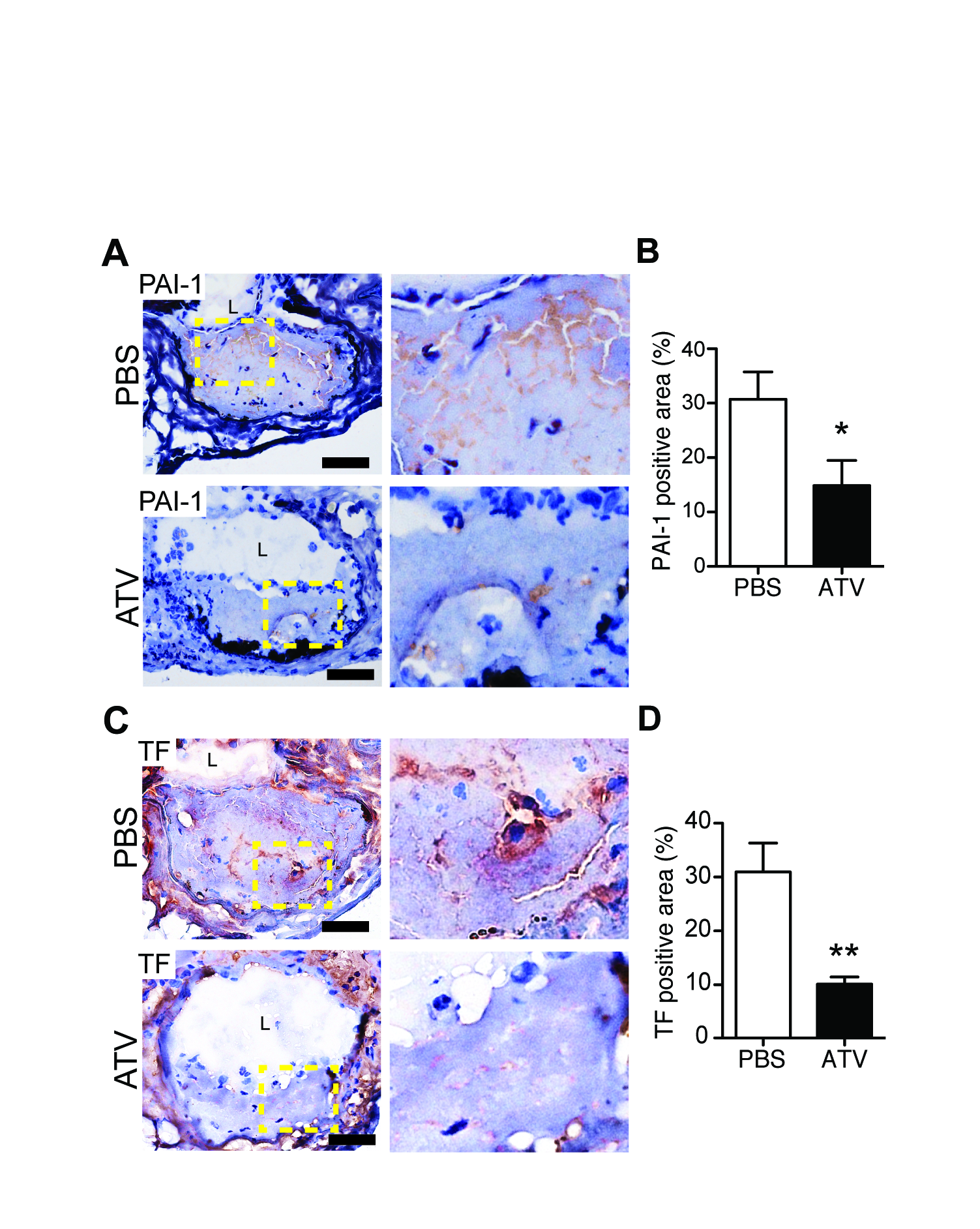

Supplement: S3 Fig — Immunohistochemical analysis of thrombosis sections shows a decrease in thrombus PAI-1 (A, B) and TF (C, D) in atorvastatin-treated mice compared to PBS-treated mice at day 4. Yellow dashed box marks the zoomed inset area. ATV = atorvastatin. *p<0.05; ** p<0.01. Bars represent mean±SD of n = 12 mice per group. Scale bars, 25 μm. (TIF) [file pone.0116621.s003.tif]

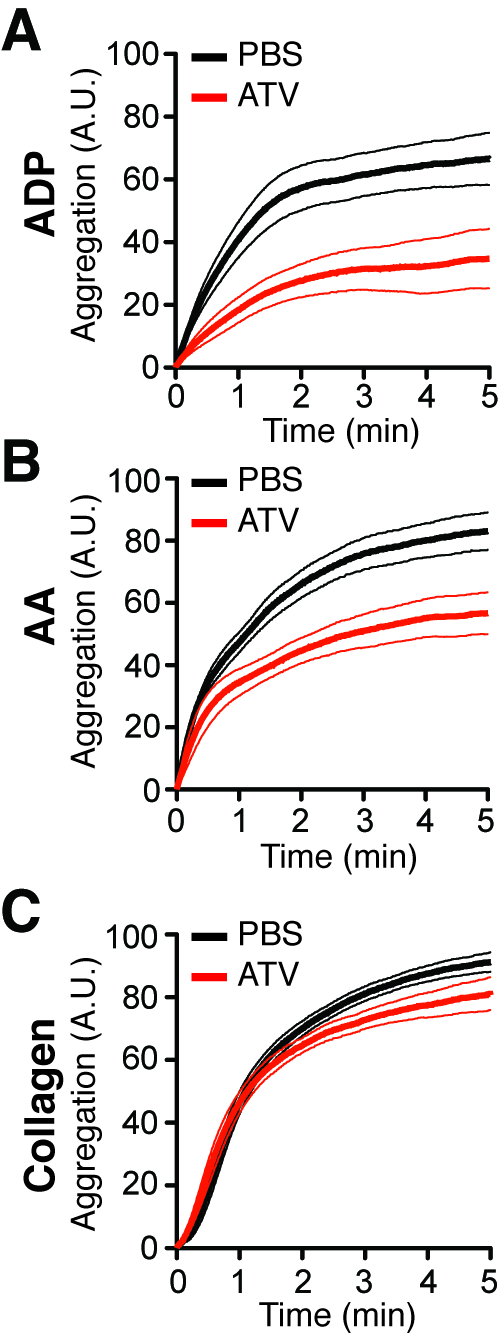

Supplement: S4 Fig — Atorvastatin (red line) decreased platelet aggregation compared to PBS-treated mice (black line). Adenosine diphosphate (ADP; A), arachidonic acid (AA; B) and collagen (C) were used to stimulate activation of platelets in pooled whole blood samples. Thick solid line and thin lines depict the mean and its SD, respectively. (TIF) [file pone.0116621.s004.tif]

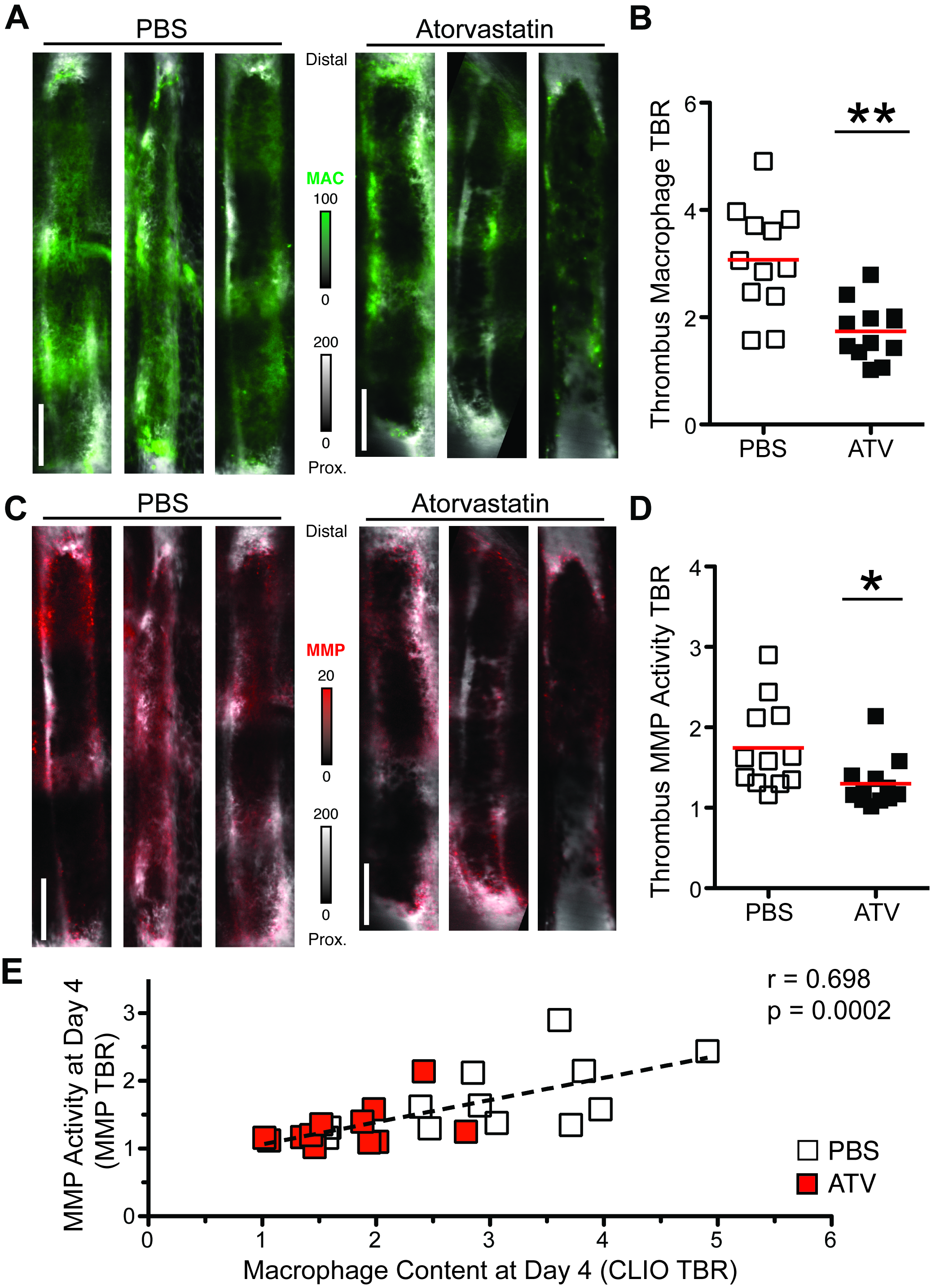

Supplement: S5 Fig — Macrophages (A) and MMP activity (C) were visualized in DVT 24 hours after intravenous injection of CLIO-AF555 (green) and MMPSense680 (red), respectively (A, C). Thrombus macrophage content (B) and MMP activity (D) were reduced in atorvastatin-treated animals compared to PBS. In vivo macrophage content and MMP activity correlated well in both statin-and PBS-treated animals (E). MAC—macrophage; MMP—matrix metalloproteinase, TBR, target-to-background ratio. *p<0.05; **p<0.01. Mean is marked by red line in scatter plots of 12 animals per group. Scale bars, 200 μm. (TIF) [file pone.0116621.s005.tif]

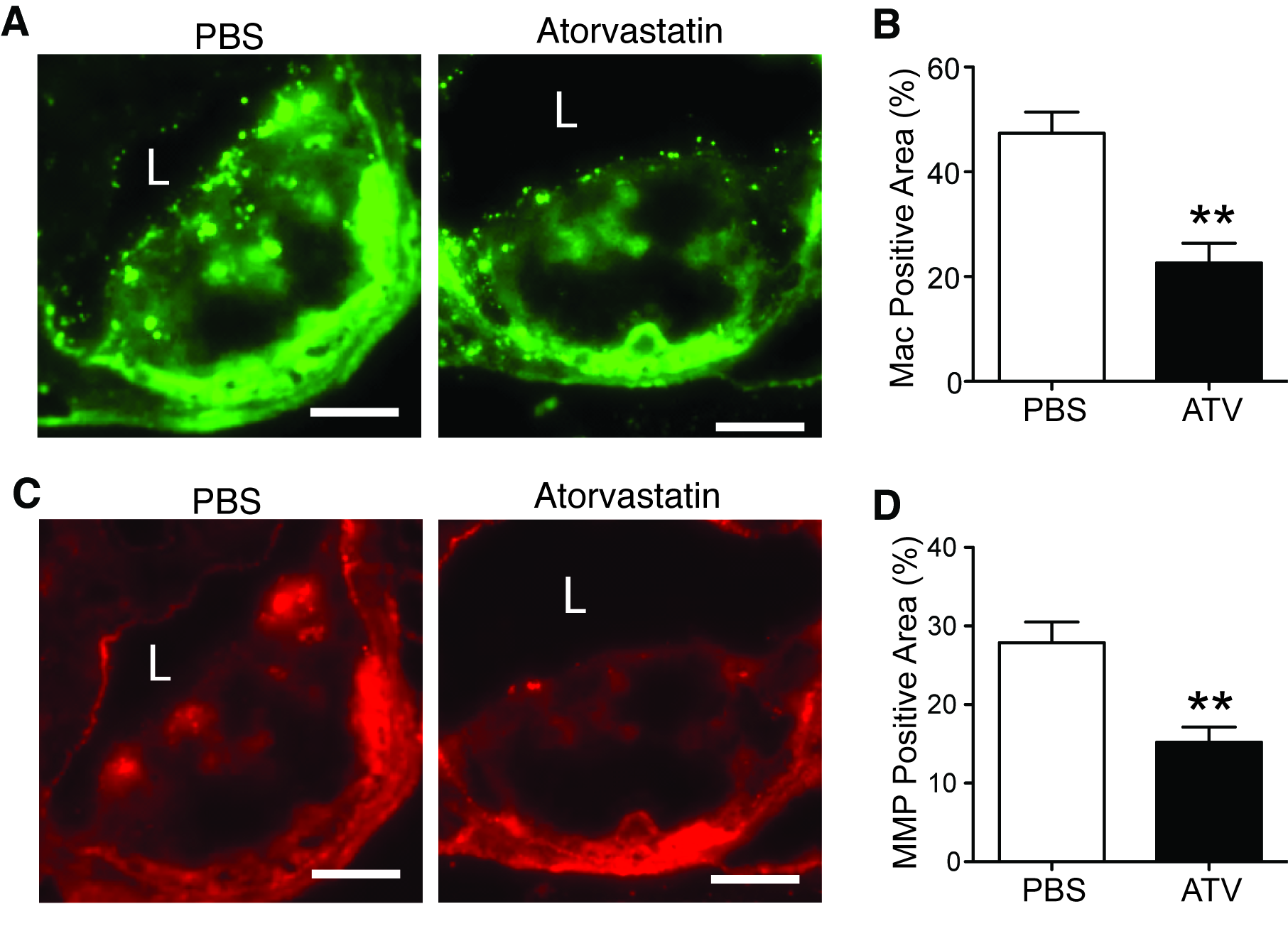

Supplement: S6 Fig — FM shows reduced macrophage content (A, B; green) and MMP activity signals (C, D; red) in statin-treated compared to PBS-treated animals. Bars represent mean±SD of 12 animals per group, average of three section per animal. Scale bar, 25 μm. (TIF) [file pone.0116621.s006.tif]
